# Kids Really Are Just Small Adults: Utilizing the Pediatric Triangle with the Classic ABCD Approach to Assess Pediatric Patients

**DOI:** 10.7759/cureus.7424

**Published:** 2020-03-26

**Authors:** Ayanna Walker, Andrew Hanna

**Affiliations:** 1 Emergency Medicine, University of Central Florida College of Medicine/Hospital Corporation of America Graduate Medical Education Consortium of Greater Orlando, Orlando, USA; 2 Emergency Medicine, Osceola Regional Medical Center, Orlando, USA; 3 Emergency Medicine, University of Central Florida, Orlando, USA

**Keywords:** pediatrics, emergency medical services, pediatric assessment triangle, critical care, emergency medicine, acute care

## Abstract

Pediatric altered mental status is a commonly feared presentation of children in both the emergency department and prehospital setting. Given the usual difficulties we face when treating children, utilizing systematic tools can help us to remain thorough and consistent when evaluating any given child. By using a standardized method of evaluation and diagnosis, first responders and emergency physicians can set aside their worries of mismanagement and provide adequate care to children with undifferentiated altered mental status. This article reviews one of the many approaches toward assessing children's clinical status, walks through the elements that comprise it, and examines how it can be used to make emergency providers more confident.

## Introduction and background

The practice of emergency medicine requires a broad range of knowledge. Both paramedics and emergency medicine physicians have subject areas they enjoy and perform well in. Many enjoy the adrenaline rush associated with taking care of a patient in cardiac arrest or stabilizing a trauma victim. However, when it comes to attending to pediatric emergencies, most of them are susceptible to self-doubt and suddenly become gun-shy. In this paper, we review the primary assessment for pediatric treatment and highlight the treatment of ventilation, oxygenation, and circulatory abnormalities that could prevent the deterioration to cardiopulmonary arrest in children. Using a systematic approach and recognizing the similarities between the adult and pediatric primary assessment will allow the provider to feel more comfortable when taking care of these patients.

Although studies suggest that acute care providers are comfortable with the management of pediatric patients, the anecdotal consensus among prehospital and emergency providers suggests that treating critically ill children can be a frightening experience. In critical care assessments, providers often rely on information provided by the patient to help guide treatment. Adult patients can express how they feel about the nature of their injury and help the provider understand their mental state by providing answers to a few short questions. As children are often unable to assist in their own assessment, there is far more pressure on the provider to independently evaluate and dictate correct treatment options for the ill child. Many institutions and organizations have attempted to provide quick, objective, and standardized tools to assist with this, such as the Yale Observation Scale, the Pediatric Glasgow Coma Scale, the Pediatric Trauma Score, the Pediatric Risk of Mortality score, and the one which is almost universally followed: the Pediatric Assessment Triangle [1-5].

## Review

**Figure 1:**
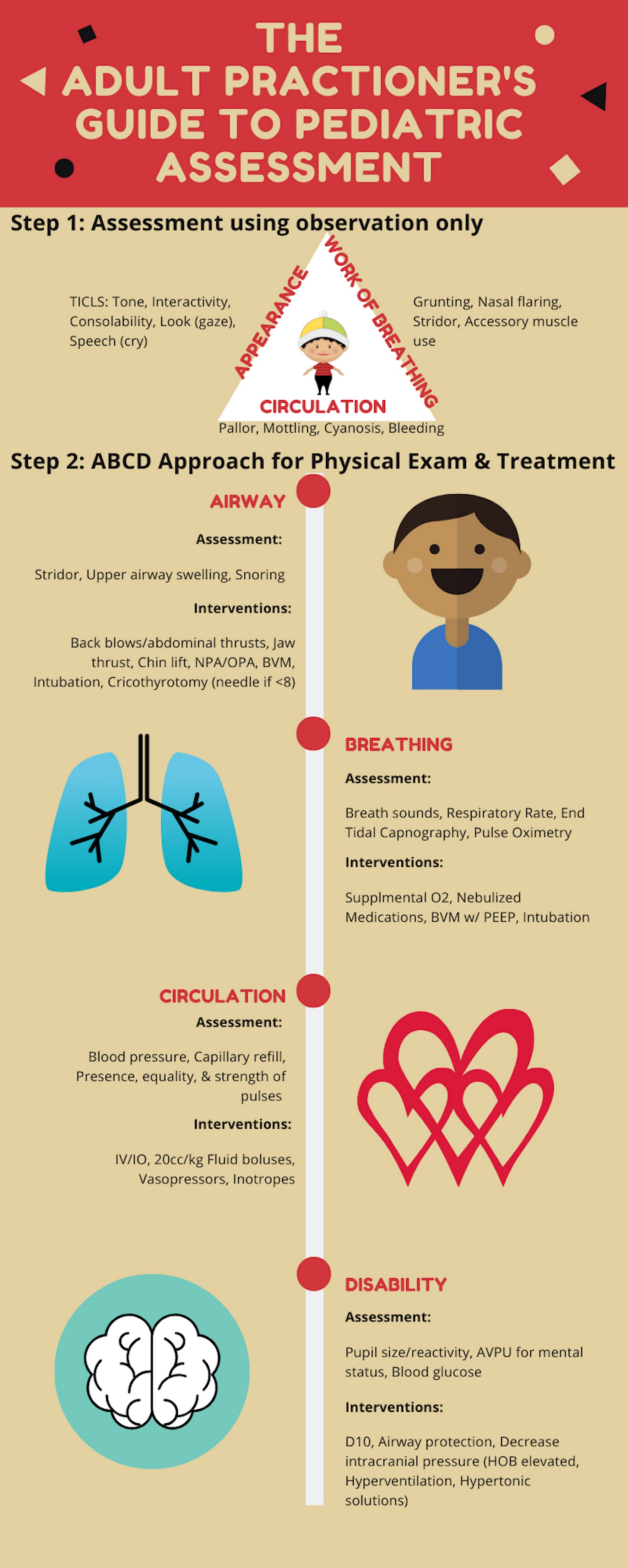
The adult practitioner's guide to pediatric assessment NPA: nasopharyngeal airway; OPA: oropharyngeal airway; BVM: bag-valve-mask; PEEP: positive end-expiratory pressure; AVPU: alert, verbal, pain, unresponsive; HOB: head of bed

In this study, we elaborate on the approach of using the Pediatric Assessment Triangle as a tool for simple observation in pediatric treatment (Figure 1).

For the most part, this assessment does not have to involve touching the children, thereby allowing information to be garnered without causing any inconvenience to them. This approach can usually be performed within 30 seconds. Following the general assessment using the Pediatric Assessment Triangle, we use the well-known ABCD approach, just as we do in adult medicine, to continue with our observational assessment, now incorporating the physical exam, objective measurements, and life-saving time-sensitive treatments.

Programs such as the Pediatric Advanced Life Support (PALS), Advanced Pediatric Life Support (APLS), and Emergency Nursing Pediatric Course (ENPC) have all endorsed the use of the Pediatric Assessment Triangle [6-8]. It is a helpful way to identify which kids are sick. Every encounter with a pediatric patient should start with a general assessment that focuses on observation. This initial assessment does not require much physical contact with the child.

Each side of the Pediatric Assessment Triangle represents an area of assessment: appearance, work of breathing, and circulation. Many utilize the triangle simply to remember these key areas. Taken a step further, if we shade in the side(s) of the triangle with the abnormal findings, we can create a table that gives a differential (Table 1).

**Table 1:** Components of the Pediatric Assessment Triangle and general impressions gathered

Differentials	Appearance	Work of breathing	Circulation
Stable	Normal	Normal	Normal
Respiratory distress	Normal	Abnormal	Normal
Respiratory failure	Abnormal	Abnormal	Normal
Shock	Normal or abnormal	Normal	Abnormal
Central nervous system/metabolic disturbance	Abnormal	Normal	Normal
Cardiopulmonary failure	Abnormal	Abnormal	Abnormal

The differentials are broad and include the following parameters: stable, respiratory distress, respiratory failure, shock, central nervous system/metabolic issues, and cardiopulmonary failure. As an example, if the child only has an issue with work of breathing, the chart points us to respiratory distress, whereas if there is an issue with work of breathing and appearance, it is likely to be respiratory failure.

There are many similarities between the adult and pediatric assessment with regard to two of the three categories in the Pediatric Assessment Triangle: work of breathing and circulation. However, appearance is very specific to pediatrics. For this reason, it is helpful to remember the mnemonic TICLS. It stands for tone, interactivity, consolability, look (gaze), and speech (cry). When it comes to assessing the appearance of the child, it becomes important to observe their tone. We will ask ourselves, “are they limp or are they stiff?” We will also be observing their interaction with their caregivers. Do they make eye contact? Are they tracking? Are they consolable? Lastly, for appearance, we are listening for the pitch of their cry and, if they are old enough to speak, we are observing their speech patterns.

The second side of the Pediatric Assessment Triangle is work of breathing. Again, most of this part of the assessment can be performed without physically touching the child. Work of breathing includes listening for audible breath sounds and observing for signs of increased work of breathing. For this part of the assessment, since we are trying to limit any physical interaction with the child, we are not going to use a stethoscope, but are only listening for stridor and grunting, as these can be heard without one. Signs that indicate increased work of breathing include nasal flaring, abdominal retractions, suprasternal retractions, and scalene muscle use.

The last side of the Pediatric Assessment Triangle is circulation. Again, trying to minimize physical interaction with the child during this initial assessment, we will be observing for pallor, mottling, cyanosis, and bleeding.

The Pediatric Assessment Triangle offers the ability to decide quickly if a child is sick. It also gives providers a systematic approach and relies less upon experience, vigilance, and repetition, which is often difficult for those who work both in an adult and pediatric setting. After determining whether the child is sick, which will help us in determining what resources we need to mobilize, the ABCD approach can be used to further assess the child. This will now incorporate physical exam findings, objective measurements, and life-saving treatments. What is helpful about the ABCD approach is that it is generalizable to both adults and pediatrics. Noticing the similarities decreases the stress level and the need for reliance on memorization.

Using the ABCD approach, we will review the assessment and life-saving interventions. A is for the airway. This includes assessing for stridor, snoring, lack of breath sounds, and upper airway swelling. Abnormalities can result from foreign body ingestion, leading to obstruction, anaphylaxis, and infections like epiglottis and retropharyngeal abscesses. If any abnormality is found during this assessment, treatment may include back blows if the child is less than a year old; abdominal thrusts if older; alligator forceps, suctioning, chin lift or jaw thrust, airway adjuncts like a nasopharyngeal airway (NPA) or oropharyngeal airway (OPA), bag-valve-mask (BVM), endotracheal intubation (ETT), or needle cricothyrotomy if less than 8 years old; or surgical cricothyrotomy if older. It is important to note that if an abnormality is found while evaluating the airway, we must not continue down the assessment pathway without airway intervention.

After addressing the life-threatening conditions during the airway assessment, we can move on to assessing B for breathing. Using the stethoscope, we will be evaluating for breath sounds such as crackles and wheezing, which may indicate conditions like pneumonia, obstructive airway disease, and pulmonary edema. Respiratory rate, end-tidal capnography, and pulse oximetry are the objective measurements obtained during this stage to add to the assessment. Treatment may include supplemental oxygen, nebulized medications, BVM, and endotracheal intubation.

Next is C, the evaluation of the child’s circulation. This involves examining the child for equality and strength of pulses bilaterally, obtaining a blood pressure, and getting capillary refill. Capillary refill is one of the most useful signs, and the normal time for it is less than 3 seconds. Anything higher may indicate shock. Treatment during this step in the evaluation may include intravenous or intraosseous access, fluid resuscitation with 20-cc/kg boluses, vasopressors, and inotropes. Dopamine used to be the pressor of choice for pediatrics, but now we are gathering data to re-evaluate this. The American Heart Association still recommends dopamine as the first-line pressor in normotensive pediatric septic shock, norepinephrine in hypotensive “warm shock”, and epinephrine in hypotensive “cold shock.”

The D in the ABCD approach involves assessing the child’s mental status, obtaining a glucose level, and checking for pupil size. For this, the AVPU scale is the most commonly used tool: A is for alert, V is for verbal response, P is for the response to pain, and U stands for unresponsive. Checking pupil size is important in assessing for increased intraocular pressure. Treatment during this step may include administering glucose, managing the airway based on decreased responsiveness leading to decreased airway protection, and methods to decrease intraocular pressure (IOP), which may include hyperventilation, the elevation of the head of the bed (HOB), and administration of hypertonic solutions.

## Conclusions

When dealing with sick children, it is important to have a systematic approach with as little memorization as possible. For those taking care of both adults and kids, merging these two assessments into one will help to retain as much information as possible. We usually talk about how kids are not just small adults, but often we do not highlight the similarities. Highlighting the similarities will allow us to simplify the overall clinical assessment approach and help us to categorize sick vs. not sick, which is the first step in the practice of emergency medicine.
